# Supplementary material for: Baseline Characteristics and Outcomes of Cancer Patients Infected with SARS-CoV-2 in the Lombardy Region, Italy (AIOM-L CORONA): A Multicenter, Observational, Ambispective, Cohort Study
Source: Cancers (Basel). 2021 Mar 16;13(6):1324. doi: 10.3390/cancers13061324 (PMC7998451; doi:10.3390/cancers13061324)
Supplement: Supplementary file 1 [file cancers-13-01324-s001.pdf]

Supplementary Materials

Baseline Characteristics and Outcomes of Cancer Patients Infected with SARS-CoV-2 in the Lombardy Region, Italy (AIOM-L CORONA): A Multicenter, Observational, Ambispective, Cohort Study

Serena Di Cosimo, Barbara Tagliaferri, Daniele Generali, Fabiola Giudici, Francesco Agustoni, Antonio Bernardo, Karen Borgonovo, Gabriella Farina, Giovanna Luchena, Andrea Luciani, Franco Nolè, Laura Palmeri, Filippo Pietrantonio, Guido Poggi, Paolo Andrea Zucali, Emanuela Balletti, Giovanna Catania, Ottavia Bernocchi, Federica D'Antonio, Monica Giordano, Francesco Grossi, Angioletta Lasagna, Nicla La Verde, Mariangela Manzoni, Benedetta Montagna, Angelo Olgiati, Alessandra Raimondi, Irene Rampinelli, Elena Verri, Alberto Zaniboni, Massimo Di Maio, Giordano Beretta and Marco Danova

Table S1. Characteristics of 29 non metastatic patients not receiving SACT.

Characteristic	Patients (*N* = 29)
**Median age (Range), years**	68 (45–85)
**Male sex (N,%)**	19 (65.5%)
**Comorbidity (N, %)**	
None	7 (24.1%)
1	10 (34.5%)
≥2	12 (41.4%)
**ECOG Performance Status (N, %)**	
0	17 (81.0%)
1	3 (14.3%)
2–3	1 (4.7%)
Subtotal	21
Unknown (N,%)	8 (27.6%)
**Tumour diagnosis (N, %)**	
Colorectal	8 (27.6%)
GI-no colorectal	7 (24.1%)
Breast	6 (20.7%)
Genitourinary tract cancer	3 (10.3%)
Lung	2 (6.9%)
Head and Neck	2(6.9%)
Gynecological	1(3.4%)
**Stage (N, %)**	
I	12 (41.4%)
II	6 (20.7%)
III	11 (37.9%)
**Symptoms (N, %)**	
**Yes**	29 (100.0%)
**Type of Symptom (N, %)**	
Fever	22 (75.9%)
Cough	16 (55.2%)
Dyspnoea	18 (62.1%)
Desaturation	18 (62.1%)
**Interstitial pneumonia (N, %)**	
Yes	23 (92.0%)
Subtotal	25
Unknown (N, %)	4 (13.8%)
**Covid 19 Therapy**	
Yes	23 (79.3%)
**Progression Disease at COVID19 diagnosis**	
Yes	0(0.0%)
Subtotal	19
Unknown	10 (34.5%)

**Oncological Progression Disease after COVID19 infection (N, %)**	
Yes	1 (4.5%)
Subtotal	22
Unknown (N, %)	7 (24.1%)
**Ordinary Hospitalization**	
Yes	24 (82.8%)
**Admission to Intensive Care Unit after Ordinary Hospitalization**	
Yes	3 (11.5%)
Subtotal	26
Unknown	3 (10.3%)
**Mortality within 30 days of COVID-19 diagnosis**	
**Yes**	9 (31.0%)

Table S2. Characteristics of 43 metastatic cancer patients not receiving SACT.

Characteristic	Patients (*N* = 43)
**Median age (Range), years**	71 (40–89)
**Male sex (N,%)**	24 (55.8%)
**Comorbidity (N, %)**	
None	11 (25.6%)
1	15 (34.5%)
≥2	17 (39.5%)
**ECOG Performance Status (N, %)**	
0	12 (36.4%)
1	16(48.5%)
2–3	5 (15.1%)^
Subtotal	33
Unknown (N,%)	10 (23.3%)
**Tumour diagnosis (N, %)**	
**Lung**	14 (32.6%)^
GI-no colorectal	12 (27.9%)
**Hematological**	6 (14.0%)^
Breast	4 (9.3%)
Colorectal	3 (7.0%)
Genitourinary tract cancer	2 (4.7%)
Melanoma	1(2.3%)
Brain	1 (2.3%)
**Symptoms (N, %)**	
**Yes**	38 (88.4%)
**Type of Symptom (N, %)**	
Fever	30 (71.4%)
Cough	11 (25.6%)
Dyspnoea	28 (66.7%)
Desaturation	30 (69.8%)
**Interstitial pneumonia (N, %)**	
Yes	23 (88.9%)
Subtotal	36
Unknown (N, %)	7 (16.3%)
**Covid 19 Therapy**	
Yes	37 (88.1%)
Subtotal	42
Unknown	1 (2.3%)
**Progression Disease at COVID19 diagnosis**	
Yes	6 (25.0 %)
Subtotal	24
Unknown	19 (44.2%)
**Oncological Progression Disease after COVID19 infection (N, %)**	
Yes	1 (3.7%)
Subtotal	27
Unknown (N, %)	16 (37.2%)
**Ordinary Hospitalization**	
Yes	40 (93.0%)
**Admission to Intensive Care Unit after Ordinary Hospitalization**	
Yes	1 (2.4%)
Subtotal	41
Unknown	2 (4.7%)
**Mortality within 30 days of COVID-19 diagnosis**	
Yes	28 (66.7%)
Subtotal	42
Unknown	1 (2.3%)

^As compared to patients on active therapy, untreated cases were more likely to present with ECOG 2–3, i.e., 5/33 (15%) versus 6/75 (8%), $p = 0.26$, lung cancer, i.e., 14/43 (32.5%) versus 21/108 (19.4%), $p = 0.08$, and haematological disease, i.e., 6/43 versus 5/108, $p = 0.04$.

Table S3. Characteristics of 61 patients not receiving anti-COVID-19 therapy.

Characteristic	Patients (*N* = 61)
**Median age (Range), years**	61 (31–88)
**Male sex (N,%)**	20 (32.8%)
**Comorbidity (N, %)**	
None	24 (39.3%)
1	28 (45.9%)
≥2	9 (14.8%)
**ECOG Performance Status (N, %)**	
0	36(76.6%)
1	7 (14.9%)
2–3	4 (8.5%)
Subtotal	47
Unknown (N,%)	14 (23.0%)
**Stage**	
I	8 (13.1%)
II	9 (14.8%)
III	6 (9.8%)
IV	38 (62.3%)
**Tumour diagnosis (N, %)**	
Breast	28 (45.9%)
GI-no colorectal	9 (14.8%)
Colorectal	7 (11.5%)
Lung	6 (9.8%)
Genitourinary tract cancer	3 (4.9%)
Head and Neck	2 (3.3%)
Melanoma	2 (3.3%)
Hematologic	2 (3.3%)
Brain	1 (1.6%)
Gynecological	1 (1.6%)
**Active Anticancer Treatment (N,%)**	
**Yes**	49 (81.7%)
**Subtotal**	60
**Unknown**	1 (1.6%)
**Symptoms (N, %)**	
**Yes**	34 (55.7%)
**Type of Symptom (N, %)**	
Fever	33 (55.9%)
Subtotal	59
Unknown	2 (3.3%)
Cough	24 (42.1%)
Subtotal	57
Unknown	4 (6.6%)
Dyspnoea	12 (21.0%)
Subtotal	57
Unknown	4(6.6%)
Desaturation	3 (5.3%)
Subtotal	57
Unknown	4 (6.6%)
**Interstitial pneumonia (N, %)**	
Yes	21 (48.8%)
Subtotal	43
Unknown (N, %)	18 (29.5%)
**Progression Disease at COVID19 diagnosis**	
Yes	6 (25.0 %)
Subtotal	24
Unknown	19 (44.2%)
**Oncological Progression Disease after COVID19 infection (N, %)**	
Yes	1 (3.7%)
Subtotal	27
Unknown (N, %)	16 (37.2%)
**Ordinary Hospitalization**	
Yes	12 (20.3%)
Subtotal	59
Unknown	2 (3.3%)
**Admission to Intensive Care Unit after Ordinary Hospitalization**	
Yes	0 (0.0%)
Subtotal	59

Unknown	2 (3.3%)
**Mortality within 30 days of COVID-19 diagnosis**	
Yes	12 (21.4%)
Subtotal	56
Unknown	5(8.2%)

© 2021 by the authors. Licensee MDPI, Basel, Switzerland. This article is an open access article distributed under the terms and conditions of the Creative Commons Attribution (CC BY) license (http://creativecommons.org/licenses/by/4.0/).
